# The "Lachnanthes Cure" of Consumption

**Published:** 1902-01-04

**Authors:** 


### THE "LACHNANTHES CURE" OF CONSUMPTION.

A somewhat amusing sequel to the prolonged correspondence on the "lachnanthes cure" of consumption with which the *Times* newspaper has been entertaining its readers during the past few months, comes in the form of a paper from the pen of Mr. M. Anslow Alabone,[1] son of Mr. Edwin W. Alabone, describing the treatment on which Colonel Le Poer Trench appears to have set such store as to have offered a large sum in order that its virtues might be investigated; a treatment which, as Mr. Anslow Alabone says, "has been considered for over a quarter of a century a secret remedy." The first thing that Mr. Anslow Alabone does is to throw overboard the lachnanthes, saying that the treatment "does not entirely depend upon lachnanthes, but is also a special mode of inhalation." In regard to lachnanthes, as to which so much has been claimed by Colonel Trench, Mr. Anslow Alabone remarks: "I cannot say I advocate such a high standard for the drug as some. . . . But I use it, having naturally been conversant with my father's treatment for many years, and seeing the number of cases he quotes as cured." "The principal agent of this treatment," he adds, "I consider is the compound comminutor," an instrument which, from the illustration given, appears to be of a somewhat formidable appearance, but the whole aim and object of which is the production of a fine spray of the various drugs employed by means of air or a mixture of air and oxygen. This apparatus is no doubt ingenious, but it is clear that its whole and sole use is to produce a fine spray, a task which can be performed in many ways according to the taste of the instrument maker, and has been performed in many ways as far back as we can remember. Still, we have nothing to say against this or any other spray producer; the question is, Can consumption be cured by inhaling sprays?

Now for what Mr. Alabone puts in the bottles of his inhaler; for if this secret remedy is not lachnanthes, and if the apparatus which is the principal agent in the treatment is but a spray producer, the virtues evidently must lie in the prescriptions. Prescription No. 1 consists of formic aldehyde, menthol, eucalyptus, and mollueine. The latter we are told is practically a glycerine of a lower specific gravity than the British Pharmacopœia product. No. 2 consists of lachnanthes, creasote, pinus canadensis, and viscidine, which is a preparation of hydrocarbon oil. No. 3 of vinum ipecac., vin. antimon., guiac. carb., and R. benz. co. No. 4 of pix. liq., terebinth, balsam peru., and R. benz. co.; No. 5 of thymol, eucalyptol, menthol, and viscidine; No. 6 of R. lachnanthes, pinus canadensis, calendulæ, and viscidine; No. 7 of eucalyptol, menthol, ol. ricini, and viscidine; No. 8 consists of terebinth, R. hamamelidis, and mollueine. Then we are told the proportions in which these bottles are combined, and it is stated that in case of hæmorrhages preparations containing ergot and terebinth, or hamamelidis and terebinth, or hamamelidis and renaglandin, are given, combined with internal preparations if the case be severe. Mr. Alabone generally prescribes a syrup containing lachnanthes, with one or more of the following:—Calcium hypophosphite, sodium hypophosphite, potassium hypophosphite, iron hypophosphite, manganese hypophosphite, quinine hypophosphite. This syrup is usually taken morning and evening, and "during the middle hours of the day I treat the most prominent symptoms." Diet is to be most nourishing and most liberal in quantity and "King's emulsion" is to be taken with meals. The patient is to be out in the fresh air all he can, if dry, and he is to sleep with his upper window down. All of this is very interesting, as describing the sort of drugs commonly employed by Mr. Alabone, or anyone else in the treatment of so variable a disease as consumption, but we look in vain for the great remedy, the hidden secret, the wonderful process which, according to Colonel Trench, the profession has suppressed. Of throat sprays there is no end. They have been used for years in every conceivable variety, while hypophosphites have been given till we are tired of them; yet here we find our old friends ipecac. and creasot, menthol and eucalyptol, and the rest all trotted out again as if they were not common property—so common as to have been long since discarded as means of cure. Poor Colonel Trench! What a collapse After all his demands for investigation this is all there is to investigate! We are reminded of what Dickens calls "the memorable and tremendous words" of Betsy Prig when, speaking of the mythical Mrs. Harris, she remarked, "I don't believe there's no sich a person."

[1] British Medical Journal, D c. 28.

